# Direct differentiation of tonsillar biopsy-derived stem cells to the neuronal lineage

**DOI:** 10.1186/s11658-021-00279-4

**Published:** 2021-08-18

**Authors:** Michal Arad, Robert A. Brown, Raju Khatri, Rodney J. Taylor, Michal Zalzman

**Affiliations:** 1 Department of Biochemistry and Molecular Biology, University of Maryland School of Medicine, 108 N. Greene Street, Baltimore, MD 21201 USA; 2 The Center for Stem Cell Biology and Regenerative Medicine, University of Maryland School of Medicine, Baltimore, MD 21201 USA; 3 Marlene and Stewart Greenbaum Cancer Center, University of Maryland School of Medicine, Baltimore, MD 21201 USA; 4 Department of Otorhinolaryngology-Head and Neck Surgery, University of Maryland School of Medicine, Baltimore, MD 21201 USA

**Keywords:** Tonsil-derived multipotent stem cells (T-MSC), Biopsies, Neuron-like cells, Neuroblasts, Neuronal stem cells (NSC), Differentiation

## Abstract

**Background:**

Neurological disorders are considered one of the greatest burdens to global public health and a leading cause of death. Stem cell therapies hold great promise for the cure of neurological disorders, as stem cells can serve as cell replacement, while also secreting factors to enhance endogenous tissue regeneration. Adult human multipotent stem cells (MSCs) reside on blood vessels, and therefore can be found in many tissues throughout the body, including palatine tonsils. Several studies have reported the capacity of MSCs to differentiate into, among other cell types, the neuronal lineage. However, unlike the case with embryonic stem cells, it is unclear whether MSCs can develop into mature neurons.

**Methods:**

Human tonsillar MSCs (T-MSCs) were isolated from a small, 0.6-g sample, of tonsillar biopsies with high viability and yield as we recently reported. Then, these cells were differentiated by a rapid, multi-stage procedure, into committed, post-mitotic, neuron-like cells using defined conditions.

**Results:**

Here we describe for the first time the derivation and differentiation of tonsillar biopsy-derived MSCs (T-MSCs), by a rapid, multi-step protocol, into post-mitotic, neuron-like cells using defined conditions without genetic manipulation. We characterized our T-MSC-derived neuronal cells and demonstrate their robust differentiation in vitro.

**Conclusions:**

Our procedure leads to a rapid neuronal lineage commitment and loss of stemness markers, as early as three days following neurogenic differentiation. Our studies identify biopsy-derived T-MSCs as a potential source for generating neuron-like cells which may have potential use for in vitro modeling of neurodegenerative diseases or cell replacement therapies.

**Supplementary Information:**

The online version contains supplementary material available at 10.1186/s11658-021-00279-4.

## Background

Human multipotent stem cells (MSCs) [1], also known as multipotent progenitor cells (MPCs) are stem cells residing on blood vessels, and can therefore be found throughout the body [2–4]. To date, MSCs have been extracted from adult bone marrow [1, 5], adipose tissue [6], tonsils [7, 8] and dental pulp [9], as well as fetal and neonatal tissues such as amniotic fluid [10], Wharton’s Jelly [11], and umbilical cord [12–14]. However, the tissue source and harvesting procedures can significantly impact their differentiation potential and marker expression profiles [15–19].

Importantly, MSCs hold promise for the modeling and treatment of neurodegenerative diseases. Neuron-like cells have been generated from MSCs derived from bone marrow [20, 21], adipose tissue [22, 23], olfactory mucosa [24] and umbilical cord [25, 26]. Animal studies further support the therapeutic effect of MSC-derived neuroblasts and undifferentiated MSCs [27–31]. Accordingly, there are currently 462 active clinical trials [32] using MSCs for the treatment of numerous diseases, with 38 active trials focused on neurodegenerative diseases. Furthermore, efficacy and safety of MSCs-based therapies were reported for neurodegenerative disorders such as Parkinson’s disease [33], Alzheimer’s disease [34, 35], ischemic stroke [36, 37], autism spectrum disorder [38] and amyotrophic lateral sclerosis [39, 40].

However, the ideal source for autologous grafts, or for the generation of universal donor banks for the treatment of neurodegenerative diseases, is a tissue specimen that can be easily retrieved from any person at any age with minimal genetic or in vitro manipulation. MSCs possess the natural ability to differentiate and secrete factors to promote tissue healing without genetic manipulation, and they do not form teratoma tumors. Consequently, rapid procedures to harvest and differentiate MSCs to the neuronal lineage offer major advantages for neurodegenerative diseases, as these cells can be extracted from different human tissues at any age. It also allows for tissue harvesting with full consent of the patients/live donors.

To this end, we have developed a highly efficient procedure that allows us to generate multipotent stem/progenitor cells from a small sample of tonsillar biopsy [8]. This is important, as harvesting tonsillar biopsies can be done with local anesthesia and can be performed in an outpatient setting, similar to a dental procedure in a fully conscious donor. Our procedure utilizes approximately 0.6 g tonsillar biopsies to yield an average of 3.72 × 10^7^ tonsillar biopsy-derived MSCs (T-MSCs), which can be further massively expanded without the risk of teratoma tumor formation [8].

During embryonic development, the head and neck tissues, including tonsils, are formed from neural crest cells that migrate from the hindbrain to form the pharyngeal pouches. Tonsils are a rich source for MSCs [7, 8], which share an embryonic origin with, and may thus have an advantage in generating neurons [41–43]. Therefore, we sought to assess and compare the neurogenic potential of T-MSCs to bone marrow derived MSCs (BM-MSCs). Here we define for the first time the potential of our T-MSCs to efficiently differentiate into neuron-like cells in vitro. We compared the neurogenic potential of T-MSCs to that of bone-marrow derived MSCs and found a high yield of neural stem cells. We show that T-MSCs rapidly commit to the neural lineage within 3 days of neural induction. Furthermore, our findings are the first to report Nestin + /Musashi1 + /Sox1 + neural stem cells derived from tonsillar biopsies. This is important, as committed NSCs can be further expanded, while mature neurons cannot abide or survive harvesting procedures.

## Methods

### Isolation and expansion of MSCs from human tonsillar biopsies

Tonsillar biopsy-derived MSC were generated as previously described [8]. The protocol was approved with informed consent by the University of Maryland, Baltimore institutional review board (IRB protocol #HP-00062781-1) and cell lines were characterized as we previously described [8]. T-MSC lines were cultured in complete T-MSC medium: DMEM (Invitrogen) 10% FBS (Takara); 1 mM sodium pyruvate (Invitrogen), 0.1 mM non-essential amino acids (NEAA; Invitrogen), 2 mM GlutaMAX (Invitrogen), 0.1 mM beta-mercaptoethanol (Life Technologies/Gibco), and penicillin /streptomycin (50 U/50 μg/ml; Invitrogen). For all cell lines, medium was changed every 3 days and cells were routinely split every 4–7 days when 70% confluence was reached, using Accutase (EMD Millipore). Unless stated otherwise, cells from each expansion were cryopreserved, and to maintain reproducibility of differentiation all cells were used for up to eleven passages. Five donors were used in this research: pediatric donors (3–7 years old): 1 female, 1 male, and adult donors (20–35): 2 females, 1 male (Additional file 2: Table S1). Bone marrow derived MSCs (BM-MSCs) were purchased from Sigma and cultured alongside T-MSCs as described above.

### Neurosphere formation

Cells were harvested by using Accutase (EMD Millipore). To generate neurospheres, harvested T-MSCs or BM-MSCs were cultured at a density of 6 × 10^4^ cells per well in ultra-low attachment 6-well plates (Corning) in neurosphere medium containing DMEM/F12 (Invitrogen), neurobasal medium (Invitrogen), at  a 1:1 ratio, supplemented with 2 mM GlutaMAX, penicillin/streptomycin (50 U/50 μg/ml; Invitrogen), 0.5X B27 Supplement (Invitrogen), 0.5X N2 Supplement (Invitrogen), 20 ng/ml basic FGF (Biolegend) and 20 ng/ml Recombinant human EGF (Biolegend). These conditions were maintained for 72 h, after which the size and number of spheres were analyzed using ImageJ software.

### Differentiation to a neural progenitor phenotype

Following 3 days of culture, neurospheres were collected, gently dissociated by Accutase and plated in 6-well cell culture plates (Corning) or on chamber slides (Fisher Scientific) pre-coated with a layer of Poly-D-lysine (30 min, room temperature; Sigma) and a second layer of Laminin (2 h, 37° C; Sigma). The cells are then grown for 3 days in basal neuronal medium containing neurobasal medium, 1X B27 supplement, 2 mM GlutaMAX. We noticed that penicillin and streptomycin caused substantial cell death at this point of the protocol. Hence, cells were maintained instead in 100 µg/ml Ampicillin (American Bioanalytical).

### Neuroblast differentiation and promotion of neuronal maturation

On day 6 of the procedure, following 48 h of adherent culture, cells were supplemented with an enriched neuronal growth medium which included: 20 ng/ mL BDNF (Gibco), 20 ng/mL GDNF (Gibco) and 500 μM Dibutyryl cAMP (Sigma) with 100ug/mL ampicillin (American Bioanalytical). It is important to note that we found, in multiple independent experiments with at least 6 replicates, that a full media change resulted in cell death and loss of the culture by the 9^th^ day of the differentiation procedure, a phenomenon similar to that seen in primary neuron cell cultures [44]. Therefore, half of the medium was removed every 3 days and replaced with fresh enriched neuronal growth medium.

### RNA extraction and reverse transcription

Total RNA was extracted from differentiated or undifferentiated control cells using the Qiagen RNeasy mini Kit, following the manufacturer instructions. cDNA was generated by using 1 µg of total RNA by Superscript III (Invitrogen) following the manufacturer’s protocol.

### Quantitative reverse transcriptase polymerase chain reaction (qRT-PCR)

To determine the expression of neuronal differentiation related genes by real-time qPCR, 10 ng cDNA was used per well in triplicates using SYBR green (Roche) following the manufacturer’s protocol. Reactions were run on the QuantStudio 3 Real-Time PCR System (Applied Biosystems). Fold induction was calculated by the delta-delta Ct method using housekeeping genes RPLP0 or GAPDH as controls. A standard curve was made for the reference gene RPLP0 or GAPDH by serial dilutions of cDNA from 100 ng to 3.125 ng. Primers are listed in Additional file 3: Table S2.

### Cell proliferation assay

For each passage, 1 × 10^5^ T-MSCs were seeded in six well plates in triplicates. Cells were grown in sub-confluence conditions and harvested by Accutase (Millipore) every 5 days. Cells were counted and 1 × 10^4^ cells were re-seeded in 6 well plates in triplicates. On the indicated time points, cells were counted, or fixed by 4% paraformaldehyde (PFA; Alfa Aesar) and nuclei were stained by DAPI and were counted in at least 6 random fields in biological triplicates. Total cell number was calculated per each sample. The proliferation doubling time measurements were calculated as follows: T_d_ = log_2_(N_t_/N_0_), where *N*_*0*_ is the number of cells seeded at time 0, and *N*_*t*_ is the average number of cells counted at time t (days).

### Immunocytochemistry

Cells were fixed in 4% PFA (Alfa Aesar) in DPBS with 10 ug/mL sucrose for 10 min at room temperature. Cells were then blocked for 10 min at room temperature in 1% BSA, 10% fetal bovine serum, and 0.2% Tween-20 in DPBS. Primary antibodies (without Ca++, Mg++) (Additional file 4: Table S3) were incubated overnight at 4 °C in blocking solution. Following washes, slides were then incubated for 90 min at room temperature with secondary antibodies (Thermo Fisher Scientific Inc.; diluted in block solution): Alexa 488 Donkey anti mouse (1:400) and Alexa 568 Donkey anti rabbit (1:800). Nuclei were stained with DAPI or TO-PRO-3 stain. Undifferentiated T-MSCs and cells without primary antibody were used as controls. All samples were visualized under a Zeiss 510-confocal microscope, a Nikon CSU-W1 Spinning disk field scanning-confocal microscope system, or a Leica DMi8 florescence microscope.

### Surface membrane markers profiling by flow cytometry

Undifferentiated cells or differentiated neuroblasts were washed in Dulbeccos phosphate-buffered saline (DPBS) (Invitrogen), and fixed in 4% PFA in DPBS for 10 min. Cells were stained for 30 min on ice with PE-conjugated anti-CD73, Alexa-647 conjugated anti-CD90 and Alexa-488 conjugated anti-CD105 (Biolegend) diluted in block solution as above (Additional file 4: Table S3). Samples were washed twice and taken for analysis by a flow cytometer (FACS Canto II; BD Biosciences) and data were analyzed by using the FCS Express 7 software.

### Statistical analysis

Data are shown as the mean ± SEM of multiple independent experiments with multiple donors in biological replicates. Student’s t-test or one-way ANOVAs were performed for statistical analyses. A significant result in ANOVA was followed by a Fisher LSD post-hoc test. In all statistical analyses, p < 0.05 was considered statistically significant. Statistical analyses and graph generation were performed with GraphPad Prism 7.03 software.

## Results

### Differentiation of tonsillar-biopsy-derived MSCs to the neuronal phenotype

Extraction of MSCs from the bone marrow presents risk to the donor and requires hospitalization [45, 46]. Full tonsillectomy-derived MSCs (T-MSCs) were recently shown to differentiate into motor neurons [47] and Schwann cells [48]. We have recently reported small tonsillar-biopsies of less than 1 g can generate an average of over 30 million tonsillar MSCs (T-MSCs), which can then be further expanded [8]. Our T-MSCs express classical MSC markers and readily differentiate to the mesenchymal lineages, while showing significantly superior proliferative capacity in vitro compared to bone marrow derived MSCs [8]. Tonsillar biopsies can be done under local anesthesia similar to that of an outpatient dental procedure, without the need for hospitalization or general anesthetics [8]. Here we developed a procedure to directly differentiate our tonsillar biopsy-derived stem cells (T-MSCs) and assessed their potential to generate neuron-like cells.

**Fig. 1:**
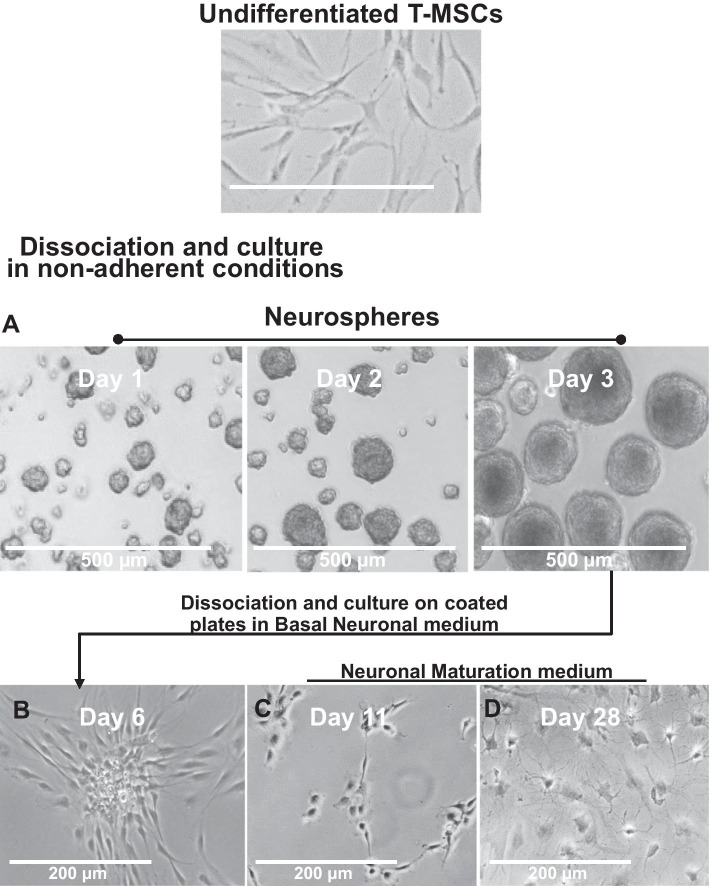
Workflow of neurogenic differentiation procedure. Undifferentiated T-MSCs are dissociated from the plate and cultured in ultra-low attachment conditions in neurosphere media. **A** Representative images of spheres after 24 h (Day 1), 48 h (Day2) and 72 h (Day 3). The resultant neurospheres are dissociated and re-plated on PDL/Laminin coated plates in basal neuronal media. **B** Representative images of cells after an additional 72 h (Day 6). On the sixth day, media is replaced with neuronal maturation medium and cells are further differentiated through Day 28. **C** Representative images of cells after a total of 11 days and **D** 28 days. These results were obtained from at least 5 donors in triplicates in multiple independent experiments.

First, T-MSCs are seeded in non-adherent conditions in ultra-low attachment plates and incubated for 72 h in neurosphere medium to develop into large floating spheres (Fig. 1A). Next, the resultant spheres are dissociated, and the cells are then seeded adherently on poly-d-lysine and laminin coated plates and are allowed to further differentiate in basal neuronal medium. On the sixth day, BDNF, GDNF and dibutyryl cAMP are added to induce further neuronal differentiation. As seen in Fig. 1B, the cells at this stage are much smaller and by day 11 of the procedure (Fig. 1C) acquire a morphology similar to neuroblasts. When cells are grown for up to 28 days, further morphologic changes and neurite-like projections from each cell and cell-to-cell contacts are observed (Fig. 1D).

### T-MSCs differentiate to neurospheres and neural stem cells with high efficiency

**Fig. 2:**
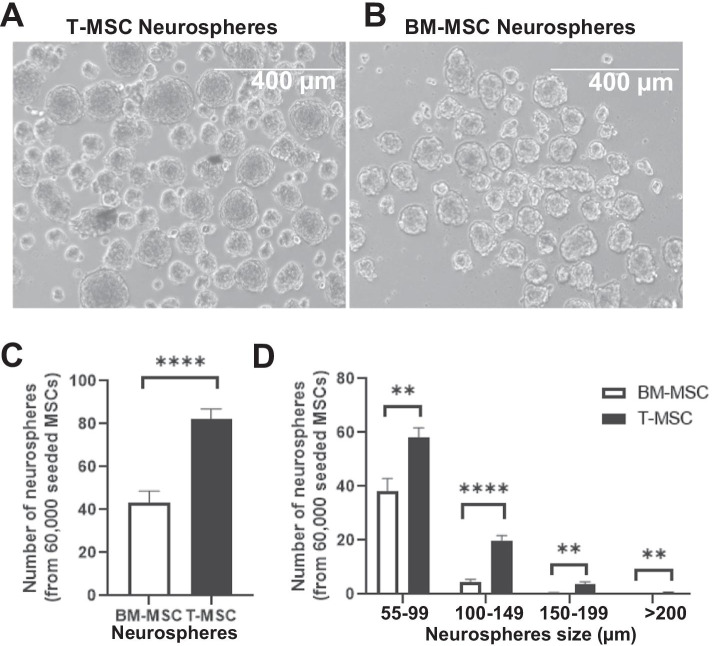
Higher efficiency of neurosphere generation in T-MSCs, compared to bone marrow derived MSCs (BM-MSCs). Comparison of MSC neurosphere formation per well of a 6 well plate in neurosphere conditions for 4 days. **A** Representative images of tonsillar biopsy derived neurospheres (T-MSC) and **B** bone marrow derived neurospheres. **C** T-MSC-derived neurosphere quantification compared to BM-MSC derived neurospheres. **D** Neurosphere quantification by size. Data shown as mean ± SEM. Asterisks signify **p < 0.01, ****p < 0.0001

The neurogenic potential of bone-marrow derived MSCs (BM-MSCs) has been previously described [20–22]. Therefore, we used BM-MSCs in our protocol alongside our T-MSCs as controls. We found that when equal number of cells were seeded in neurosphere conditions for 72 h, the T-MSC-derived spheres were larger and more numerous compared to BM-MSC-derived neurospheres (Fig. 2A, B). Our data further indicate that our T-MSCs yield on average a total 1.9-fold more neurospheres compared to BM-MSCs (p < 0.0001) (Fig. 2C). Moreover, when quantified by size groups, we found that our T-MSC-derived neurospheres yield 5.9-fold more large spheres (> 100 µm; p < 0.01) compared to those derived from BM-MSCs (Fig. 2D). A closer look at the distribution show 4.27-fold, and 8.47-fold more neurospheres of larger sizes (100–149 µm and 150–199 µm respectively), while no BM-MSC derived neurospheres were larger than 200 µm. These data suggest a higher survival and proliferation of T-MSCs in the neurosphere differentiation conditions, in turn suggesting higher neurogenic potency.

**Fig. 3:**
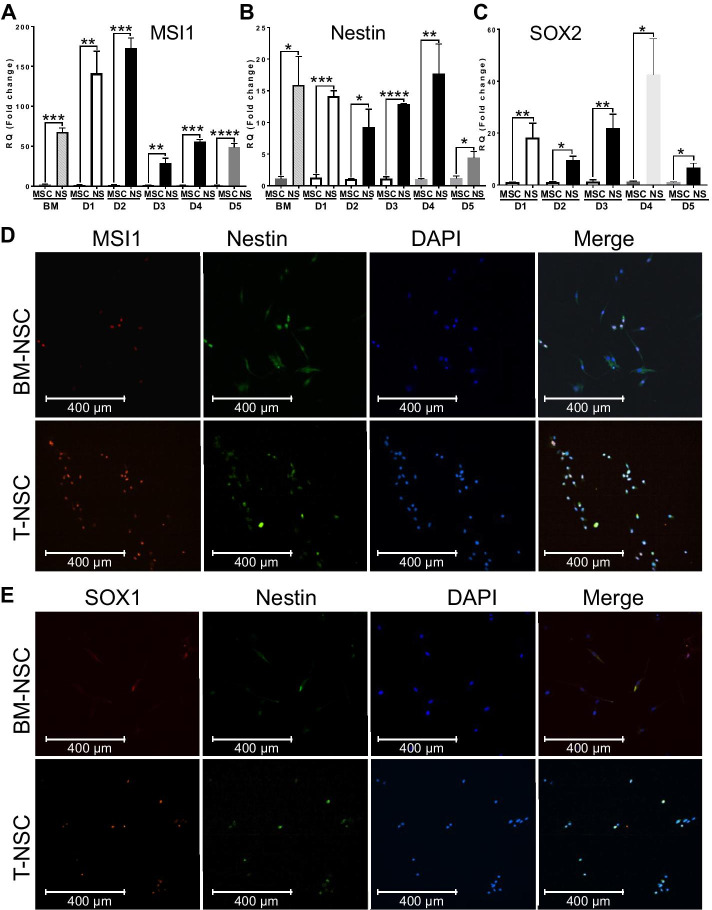
T-MSC differentiation into neuronal stem cells (NSCs). qRT-PCR from neurospheres (day-3) from 5 different donors and BM-MSCs show an increase in neural stem cell markers **A** MSI1, **B** Nestin, and **C** SOX2 compared to undifferentiated isogenic controls. Results are presented as mean ± S.E.M. obtained in triplicates; n = 5 donors in multiple independent experiments. Asterisks signify **p < 0.01, ***P < 0.001, ****p < 0.0001. **D** immunofluorescence analysis for neuronal stem cell markers in T-MSCs differentiated for 4 days to T-NSCs versus BM-NSCs in similar conditions: Nestin (green) co-stained with MSI1 (red). **E** SOX1 (red) and Nestin (green). Nuclei are stained with DAPI (blue). Undifferentiated T-MSCs and BM-MSCs were used as additional controls (see Additional file 1: Figure S1)

Next, to validate the differentiation toward a neural stem/progenitor cell (NSC) phenotype, following a 72-h incubation (day 3) T-MSCs and BM-MSCs neurospheres were collected (from 5 donors) for gene expression analyses. Our RT-qPCR data indicate a dramatic and significant upregulation of the neural stem cell markers Musashi-1 (MSI1) (Fig. 3A), Nestin (Fig. 3B), and SOX2 (Fig. 3C) across all T-MSC lines. These results were further validated by immunostaining. Neurospheres were dissociated after 72 h and plated adherently for 24 h to allow co-staining for Nestin and MSI1. Our immunostaining data confirm the upregulation of the neural stem cell (NSC) marker Nestin (green) and the expression of the NSC marker Musashi-1 (MSI1; Red) (Fig. 3D) in tonsillar-biopsy-derived NSCs. Undifferentiated T-MSCs and BM-MSCs were used as controls (Additional file 1: Fig. S1). Moreover, our data show brighter and more prevalent staining for these NSC markers when compared to BM-MSCs after differentiation in similar conditions. These data were confirmed in at least three different donors. Notably, the increase in MSI1 was also accompanied by a shift from mostly cytoplasmic to nuclear localization, indicating differentiation [49]. We further found that the increase in MSI1 and Nestin is accompanied by an increase in the NSC marker SOX1, which is co-stained within the same cells (Fig. 3E). Our results show again that the majority of our T-MSC-neurosphere-derived cells express brighter SOX1, and with higher frequency compared to BM-MSC derived neurospheres. Undifferentiated T-MSCs and BM-MSCs were used as controls for each donor tested. These data suggest that our procedure yields neural stem cells from T-MSCs following as little as 72–96 h of differentiation with high efficiency.

### Neurogenic induction into neuroblasts

Adult stem cell therapy in aged-related neuropathologies has shown promising outcomes. Transplantation of immature neural-lineage cells was shown to have better therapeutic potential than undifferentiated cells [50]. Our T-MSC-derived NSCs are characterized by several markers such as MSI1, Nestin, SOX1 and SOX2 suggesting T-MSCs may differentiate further beyond neural stem cells (Fig. 3). Thus, to promote further neuronal maturation on the third day of our procedure, the tonsil-derived neurospheres were collected, dissociated and cultured on Poly-d-lysine and laminin coated plates in a basal neuronal growth medium. On the sixth day of the protocol cells were switched to a medium we named “neuronal maturation medium” containing the factors BDNF, GDNF and Dibutyryl cAMP. Importantly, we noted that, like cultured primary neurons, after 3 days, a full media change results in cell death and loss of culture. This observation was reproducible in all donors tested. For that reason, the medium was routinely gradually changed with only half of the medium removed and replaced in each media change once every 3 days.

**Fig. 4:**
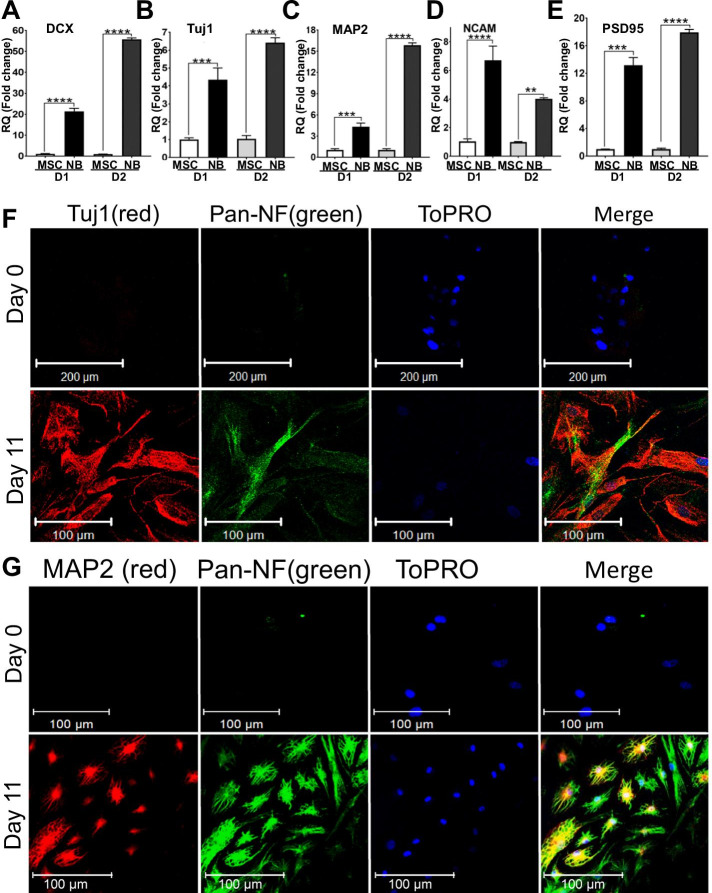
Neurogenic differentiation of T-MSCs into neuroblasts. qRT-PCR results show upregulation of the neuronal markers DCX **A** TUJ1 **B** MAP2 **C** NCAM-1 **D** and PSD95 **E** after 11 days of neuronal differentiation. 2 representative donor lines are shown. **F**, **G** Co-immunofluorescence staining show similar upregulation of neuronal markers TUJ1, Pan-Neurofilament, and MAP2 at Day 11. ToPRO serves as nuclear counterstain (blue). Data shown as mean ± SEM. Asterisks signify **p < 0.01, ***p < 0.001, ****p < 0.0001

To study the differentiation status of the cells using our neuronal induction, we assessed the upregulation of neuronal markers following 11 days of differentiation. Our data by RT-qPCR indicate that neuroblasts derived from T-MSC lines show highly upregulated DCX (doublecortin) (Fig. 4A), Tuj1 (also known as TUBB3 or β3-tubulin) (Fig. 4B), MAP2 (Microtubule Associated Protein 2) (Fig. 4C), NCAM-1 (Neural Cell Adhesion Molecule 1) (Fig. 4D) and PSD95 (Postsynaptic Density Protein 95) (Fig. 4E) compared to undifferentiated T-MSCs. These results were reproduced in at least three donors. Undifferentiated T-MSCs were used as controls. Our immunostaining data further validate a major upregulation in Pan-neurofilament (Smi312), Tuj1, and MAP2, suggesting acquisition of a neuroblast phenotype (Fig. 4F, G).

### Generation of post-mitotic, neuron-like cells

**Fig. 5:**
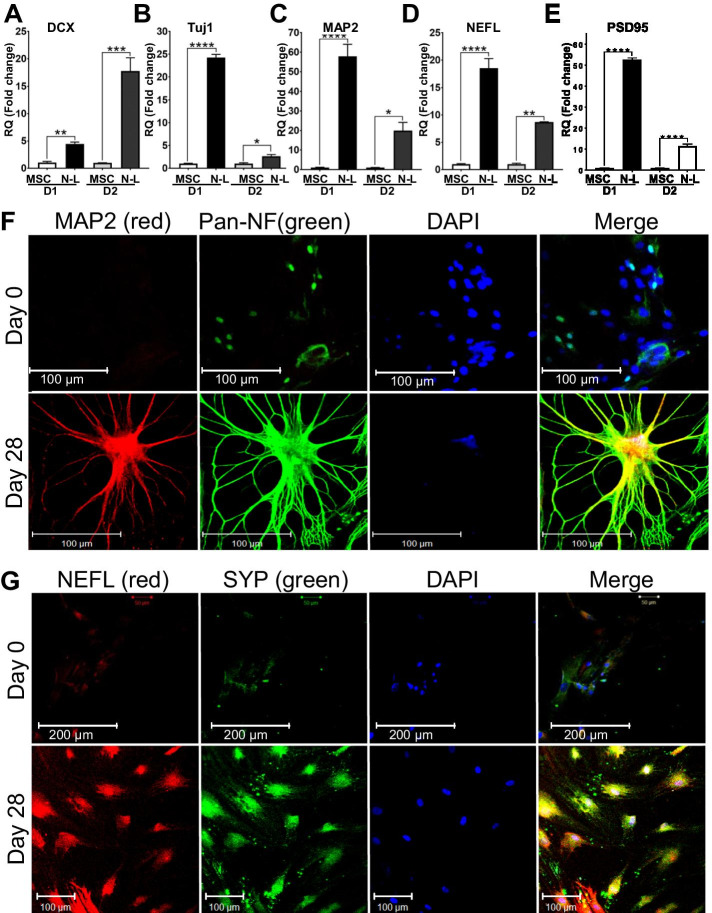
T-MSC acquire neuron-like phenotype following 28 days of differentiation. qRT-PCR results show a continued upregulation of the neuronal markers: **A** DCX, **B** Tuj1, **C** MAP2, **D** NEFL, and **E** PSD95 after 28 days of differentiation. 2 representative donor lines are shown. Result presented as mean ± S.E.M. Results were validated in n = 5 donors in multiple independent experiments. Data shown as mean ± SEM. Asterisks signify *p < 0.05, **p < 0.01, ***p < 0.001, ****p < 0.0001. **F** Consistently, co-immunofluorescence staining shows a similar upregulation of neuronal markers MAP2, Pan-neurofilament, neurofilament-L (NEFL) and **G** Synaptophysin (SYP) DAPI serves as a nuclear counterstain

To assess the neurogenic potential of our T-MSCs, we allowed the cells to further differentiate in neuronal maturation medium and assessed their differentiation after 28 days in our induction medium. Interestingly, further induction shows a reduction in the early neuroblast markers such as DCX relatively to the increase observed in day 11 neuroblasts (Fig. 5A, Fig. 4A), whereas Tuj1, MAP2, NEFL and PSD95 (Fig. 5B–E) were robustly and significantly upregulated, suggesting further neuronal maturation.

**Fig. 6:**
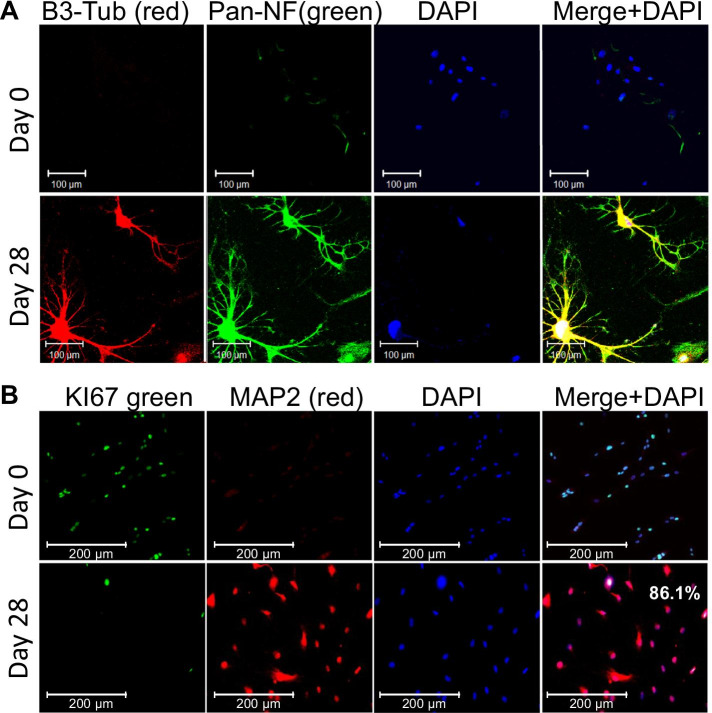
Increase in neuronal markers and downregulation of proliferative markers at Day 28. Immunofluorescence staining shows an increase in the neuronal markers **A** Tuj1 (B3-Tub) with Pan-neurofilament (Pan-NF), and **B** MAP2, while the proliferative marker KI67 decreases **B** DAPI serves as nuclear counterstain

The increase in these neuronal marker transcipts and the changes in cell morphology were further validated by immunostaining. Our data indicate that following 28 days of differentiation, our cells co-stained and were strongly positive for the Pan-neurofilament SMI312 and MAP2 (Fig. 5F), the neurofilament NEFL and the major synaptic vesicle protein p38/synaptophysin (SYP) (Fig. 5G), and further co-stained for TUBB3 (Tuj1) and Pan-Neurofilament (SMI312) (Fig. 6A). These findings are important, as these factors directly regulate the neuroblast to mature neuron transition.

Our differentiation procedure was designed to drive T-MSCs towards neuroblasts without skewing them towards a specific type of neuron lineage. However, the observed phenotypic and morphologic changes, prompted us to assess for neuronal sub-lineages. We first performed a qRT-PCR screen for neuronal sub-lineage markers. Interestingly, our results indicate, in all donors, a major upregulation of the dopaminergic markers Aromatic L-amino Acid Decarboxylase (AADC) (Additional file 1: Fig. S2A), Dopamine Active Transporter (DAT) (Additional file 1: Fig. S2B) and Nuclear Receptor Related 1 (NURR1) (Additional file 1: Fig. S2C) after 28 days of differentiation. We further observed a modest upregulation of the glutamatergic marker Vesicular Glutamate Transporter 1 (vGLUT1) (Additional file 1: Fig. S2D), while Glutaminase (GLS) was highly expressed in MSC and remained unchanged at Day 28. Other lineages were excluded as the cholinergic neuron markers Vesicular Acetylcholine Transporter (vAChT) and Choline Acetyltransferase (ChAT), the GABAergic neuron markers Glutamate Decarboxylase (GAD65) and Vesicular GABA Transporter (vGAT), and the noradrenergic neuron marker Dopamine Beta-Hydroxylase (DBH) were unchanged or undetectable.

Based on our qRT-PCR screen, we then assessed the expression of the neurotransmitters dopamine and glutamate in our cells. Immunostaining analyses show that 15% of our cells are dopamine positive and co-stained with Pan-Neurofilament (SMI312; Additional file 1: Fig. S3A). We further found that the DOPA positive cells are also co-stained with the post-mitotic neuronal marker NeuN (Additional file 1: Fig. S3B). To validate potential differentiation towards the glutamatergic lineage, cells were co-immunostained for L-glutamate (L-GLUT) and Pan-Neurofilament. Our data show 20% of the cells are L-GLUT positive (Additional file 1: Fig. S4A) and further show these cells express NeuN (Additional file 1: Fig. S4B). Taken together, these findings suggest that our protocol allows for the generation of post-mitotic neuron-like cells that express the neurotransmitters dopamine or glutamate.

### Loss of T-MSC phenotype and rapid commitment to the neuronal lineage

**Fig. 7:**
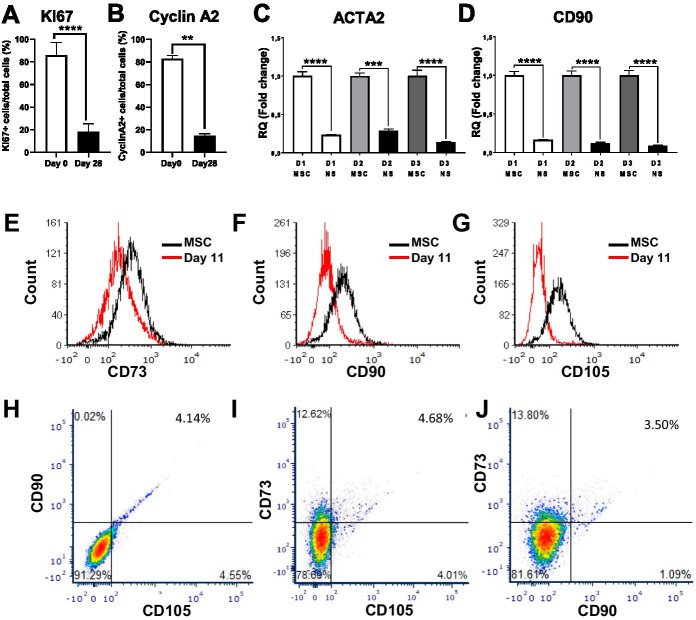
Loss of proliferation and the MSC phenotype with neurogenic differentiation. Quantification of immunostaining analyses averaged from multiple donors indicate a dramatic reduction in the replication markers **A** KI67 and **B** Cyclin A2. Asterisks signify **p < 0.01, ****p < 0.0001; n = 5 in triplicates. **C** qRT-PCR results show a major decrease of the pericyte marker ACTA2 and **D** the MSC marker CD90 after 72 h of differentiation as neurospheres. Data shown as mean ± SEM. Asterisks signify ***p < 0.001, ****p < 0.0001. Data from 3 representative donors are shown. Consistently, flow cytometry analyses of MSC markers in day 11 neuroblasts, demonstrate both low intensity and an overall decrease in positive cells for the MSC markers: **E** CD73 with 16.4%; **F** CD90 with 4.2% and **G** CD105 6.4%. **H** Flow cytometry further show that the vast majority of the cells no longer co-express the MSC markers CD90 and CD105, **I** CD73 and CD105 and **J** CD73 and CD90. Data show representative experiments from 3 different donors in biological duplicates

One important consideration in determining the differentiation status of T-MSC-derived neuron-like cells is whether and when they are committed to the neural lineage. Stem/progenitor cells committed to the neural lineage are expected to demonstrate a parallel loss of their stem cell phenotype through a decrease in proliferation and at the same time a decrease in pericyte or MSC markers, alongside the increase in neuronal lineage markers. Our data indicate that while the undifferentiated cell-population double every 36 h, the differentiated cells did not substantially proliferate and their end number was 1.5 times higher after 28 days. Therefore, to assess cell division capacity at the end of the experiment (Day 28), we used the antigen KI67, which marks proliferating cells from S phase to G2/M phase. Remarkably, our co-immunostaining analysis data indicated a marked decrease in cell replication and found that at this stage of differentiation MAP2 positive cells did not express KI67 (Fig. 6B). Furthermore, quantification of KI67 in our neuron-like cultures show only 18.2% of the cells were KI67 positive, compared to 86.1% of the undifferentiated cells (p < 0.01), (Fig. 7A). Consistently, independent experiments also show that 14.5% of the cells are Cyclin A2 positive while 82.9% are positive in the undifferentiated cells (Fig. 7B), suggesting the majority of the neuron-like cells do not replicate.

To further determine commitment to the neuronal lineage, cells were taken at early stages of the procedure and assessed for MSC/pericyte marker expression. Our qRT-PCR results demonstrate a dramatic downregulation of the pericyte marker ACTA2, and the MSC marker CD90 in neurospheres as early as three days after neural induction (p < 0.001) (Fig. 7C, D). By the eleventh day of our protocol, flow cytometry analyses demonstrate an extensive decrease in both the intensity and positive cells for the MSC markers CD73, CD90 and CD105 (Fig. 7E–G respectively). Our analyses further show that the vast majority of cells (> 95%) no longer co-express CD90/CD105 (Fig. 7H), CD73/105 (Fig. 7I) or CD73/CD90 (Fig. 7J). Taken together, our data demonstrate a rapid loss of pericyte and MSC multipotency markers with a concomitant increase in neuronal markers, suggestive of a rapid commitment to the neuronal lineage.

## Discussion

Adult stem cell therapy in neuropathologies has shown promising outcomes [33–40]. Utilization of immature neuroblast-like cells was shown to have better therapeutic outcomes than undifferentiated cells. The differentiation of mature neuron-like cells from bone marrow MSCs [21] and adipose MSCs [22] have been reported. T-MSC from full tonsillectomies however, were reported to generate Schwann-like cells when transplanted in vivo in mice [48], and were further shown to differentiate and express motor neuron markers [47]. Yet, the differentiation potential of tonsillar biopsy-derived T-MSCs to neurons remained to be characterized. Here we show for the first time, the differentiation of tonsillar biopsy-derived MSCs (T-MSCs) into post-mitotic neuron-like cells, by defined conditions and growth factors, with characterization of the cells at multiple timepoints. We further show that our novel procedure leads to a reduction and loss of stemness markers within as little as three days, suggesting a rapid commitment to the neuronal lineage.

Bone marrow (BM) derived stem cells are heavily studied for their potential use in neurodegenerative disease. We therefore assessed our NSCs from tonsillar-biopsy derived T-MSCs in comparison to those from BM-MSCs and found a superior ability to generate neurospheres both in number and size. Coupled with the impressive extraction efficiency of MSCs from tonsillar biopsies (at least five orders of magnitude compared to MSC extraction from bone marrow aspirate (8)), this suggests T-MSCs may be a rich and convenient source for MSC-based neuronal differentiation studies and potential clinical therapies.

For translational purposes, while most children have more than ample tonsillar tissue to harvest, we would anticipate that sedation would typically be required for this safe and short procedure. For the amount of tissue required (0.5 cm^3^), obtaining sufficient tissue is feasible in most adults and we have had success in adults through their sixth decade. While it is likely that many adults with small tonsils are candidates for tonsillar biopsy, we acknowledge there may be a minority of adults who may not be candidates for this approach either because of previously surgically removed tonsils or lack of tonsillar tissue volume.

To date, characterization of the neural stem cells from tonsillar-biopsy MSCs has not been reported. Consequently, our findings represent the first report of Nestin + /Musashi1 + /Sox1 + cells from MSC-derived neurospheres. This is important, as committed NSCs can be further expanded, while fully mature neurons cannot survive harvesting procedures. Further research is required to determine the potential of T-MSC-derived NSCs as a cell source for transplantation.

Our procedure was not designed to direct the NSCs toward a specified neuronal sub-type. Thus, we focused on the expression of mature neuronal markers. Our results show a dramatic change in cell morphology, as well as upregulation of MAP2, Tuj1, Neurofilaments, PSD95, and NCAM-1, suggesting that within as early as 11 days, our T-MSC-derived neuroblasts have adopted a neuroblast-like phenotype. By day 28, alongside with further morphological changes and the significant increase in neuronal markers, the cells express mature, synaptic markers such as Synaptophysin and PSD95. Finally, cell counts suggest that cells only doubled one time during the 28 days of the differentiation procedure, whereas the undifferentiated cells replicate every 36 h. We further found that over 95% of our neuron-like cells lost the co-expression of multipotency markers, yet, they express the post-mitotic neuronal marker NeuN/Rbfox3 in the nucleus by day 28, while undifferentiated cells are negative. The corresponding dramatic decrease in proliferation and in the proliferation markers KI67 and Cyclin A2 indicate the majority of the cells are no longer replicative and further suggest maturation and acquisition of a post-mitotic, neuron-like phenotype.

## Conclusions

Procedures for expansion and efficient differentiation of adult stem cells with minimal invasive manipulations are still needed for precision medicine. Previous studies have shown that MSCs from different tissue sources and the derivation processes determine the neuronal differentiation potency of MSCs. Here we report for the first time a procedure that allows the generation of a clinical-scale dose of MSCs from tonsillar biopsies, which can be extracted in a short, outpatient procedure, and efficiently achieve a high yield of neuron-like cells. Our data show that in vitro differentiation of biopsy-derived MSCs, leads to their commitment toward the neural lineage within as little as three days. Therefore, our studies suggest that tonsillar biopsies, of less than one gram of tissue, can be an excellent source of neural stem cells and neuroblast-like cells. Future studies should concentrate on determining the potential use of these cells in clinical and disease modeling applications in neuropathologies.

## Supplementary Information

**Additional file 1: Figure S1.** Minimal expression of NSC markers in MSCs. Undifferentiated T-MSCs and undifferentiated BM-MSCs show low IF staining intensity for the neural stem cell markers MSI1, Nestin, and SOX1. DAPI serves as a nuclear counterstain. **Figure S2.** Expression of dopaminergic and glutamatergic markers in our neuron-like cells. qRT-PCR data show a major increase of the dopaminergic neuron markers by day 28 Neuron-like cells: A. AADC, B. DAT, C. NURR1, and D. the glutamatergic marker vGLUT1. Data shown as mean ± SEM. Asterisks signify * p<0.05, ** p<0.01, *** p<0.001, **** p<0.0001. Data are shown from 3 representative donors (D1, D2, D3). **Figure S3.** Dopamine and neuronal markers in T-MSC derived neuron-like cells after 28 days of differentiation. A. Co-immunostaining show that dopamine (DOPA; red) is expressed in 15 % of our cells and is co-expressed with the neuronal marker Pan-Neurofilament (Smi 312; green), and B. the post-mitotic neuron marker NeuN (green). We found that the cells tend to grow in clusters in the culture well, therefore, figures show representative images of a positive area. DAPI marks nuclei (blue). Undifferentiated MSCs were used as controls (Day 0). **Figure S4.** Glutamatergic neuronal markers in T-MSC-derived neuron-like cells. A. Co-immunostaining show that L-glutamate (L-GLUT; red) is expressed in 20% of our cells and is co-stained with the the neuronal marker Pan-Neurofilament (Smi312), and B. with the post-mitotic neuron marker NeuN (green). Nuclei were counterstained by DAPI (blue). Undifferentiated MSCs were used as controls (Day 0). Images show representative positive cluster area.

**Additional file 2: Table S1.** Donor information.

**Additional file 3: Table S2.** List of Real-time RT-qPCR Primers.

**Additional file 4: Table S3.** Antibodies list.

## Data Availability

The authors declare that all data generated or analyzed during this study are included in this published article and its supplementary information files.

## Abbreviations

MSCs: Adult human multipotent stem cells
T-MSCs: Human tonsillar MSCs
NSC: Neuronal stem cells
MPCs: Multipotent progenitor cells
BM-MSCs: Bone marrow derived MSCs
bFGF: Basic fibroblast growth factor
EGF: Epidermal growth factors
BDNF: Brain derived neurotrophic factor
GDNF: Glial cell line-derived neurotrophic factor
MSI1: Musashi-1
SOX1: Sex determining. region Y)-box 1
SOX2: Sex determining. region Y)-box 2
DCX: Doublecortin
TUBB3: β3-Tubulin
MAP2: Microtubule associated protein 2
NCAM-1: Neural cell adhesion molecule 1
PSD95: Postsynaptic density protein 95
NEFL: Neurofilament light polypeptide
DOPA: Dopamine
AADC: Aromatic L-amino acid decarboxylase
DAT: Dopamine active transporter
NURR1: Nuclear receptor related 1
vGLUT1: Vesicular glutamate transporter 1
GLS: Glutaminase
vAChT: Vesicular acetylcholine transporter
ChAT: Choline acetyltransferase
GAD65: Glutamate decarboxylase
vGAT: Vesicular GABA transporter
DBH: Dopamine beta-hydroxylase
CD73: Cluster of differentiation 73
CD90: Cluster of differentiation 90
CD105: Cluster of differentiation 105
