# Supplementary material for: Direct differentiation of tonsillar biopsy-derived stem cells to the neuronal lineage
Source: Cell Mol Biol Lett. 2021 Aug 18;26:38. doi: 10.1186/s11658-021-00279-4 (PMC8371824; doi:10.1186/s11658-021-00279-4)
Supplement: Supplementary file 1 — Additional file 1: Figure S1. Minimal expression of NSC markers in MSCs. Undifferentiated T-MSCs and undifferentiated BM-MSCs show low IF staining intensity for the neural stem cell markers MSI1, Nestin, and SOX1. DAPI serves as a nuclear counterstain. Figure S2. Expression of dopaminergic and glutamatergic markers in our neuron-like cells. qRT-PCR data show a major increase of the dopaminergic neuron markers by day 28 Neuron-like cells: A. AADC, B. DAT, C. NURR1, and D. the glutamatergic marker vGLUT1. Data shown as mean ± SEM. Asterisks signify * p<0.05, ** p<0.01, *** p<0.001, **** p<0.0001. Data are shown from 3 representative donors (D1, D2, D3). Figure S3. Dopamine and neuronal markers in T-MSC derived neuron-like cells after 28 days of differentiation. A. Co-immunostaining show that dopamine (DOPA; red) is expressed in 15 % of our cells and is co-expressed with the neuronal marker Pan-Neurofilament (Smi 312; green), and B. the post-mitotic neuron marker NeuN (green). We found that the cells tend to grow in clusters in the culture well, therefore, figures show representative images of a positive area. DAPI marks nuclei (blue). Undifferentiated MSCs were used as controls (Day 0). Figure S4. Glutamatergic neuronal markers in T-MSC-derived neuron-like cells. A. Co-immunostaining show that L-glutamate (L-GLUT; red) is expressed in 20% of our cells and is co-stained with the the neuronal marker Pan-Neurofilament (Smi312), and B. with the post-mitotic neuron marker NeuN (green). Nuclei were counterstained by DAPI (blue). Undifferentiated MSCs were used as controls (Day 0). Images show representative positive cluster area. [file 11658_2021_279_MOESM1_ESM.pdf]

Additional Fig. S1

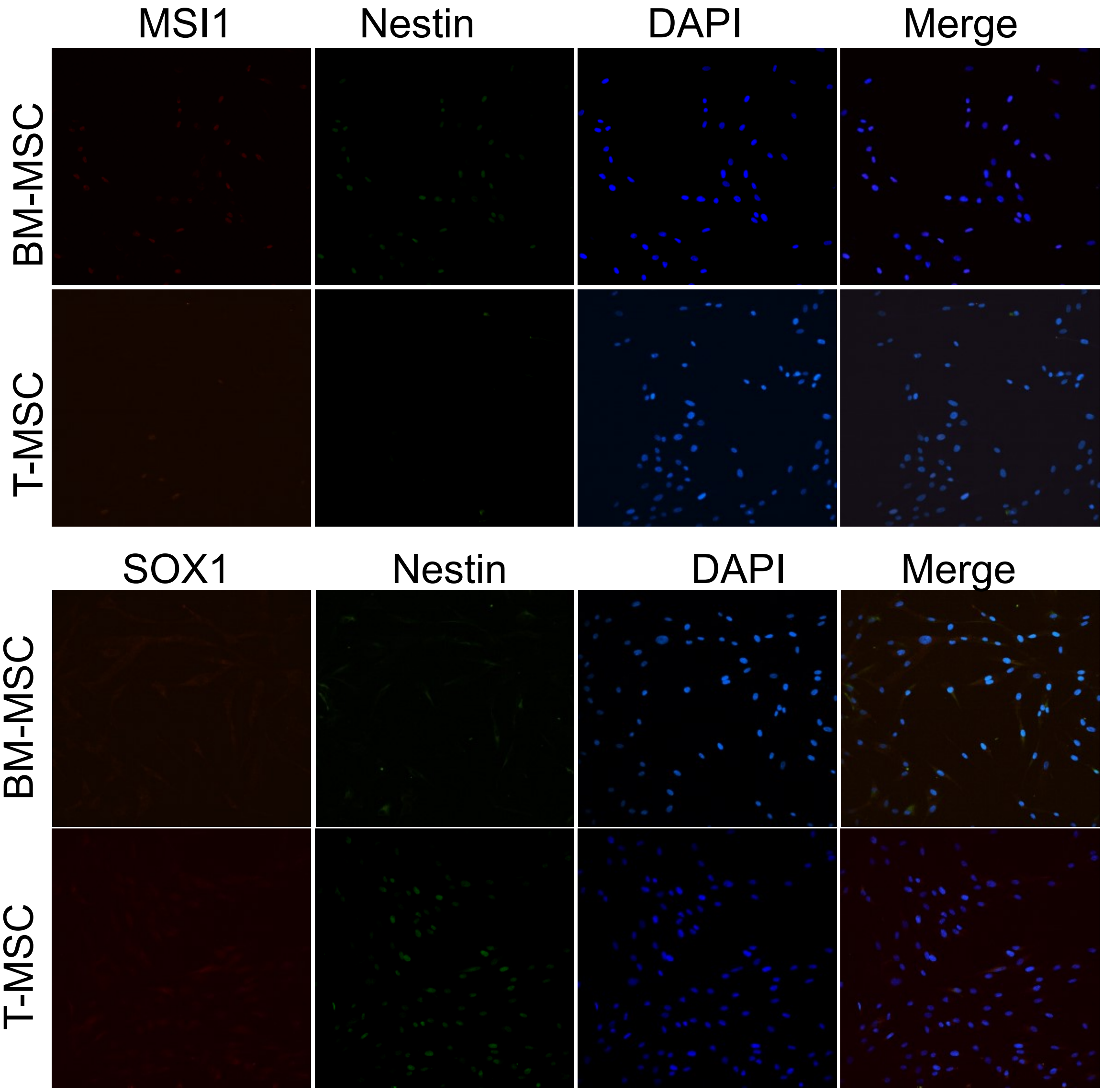

Supplementary Figure 1: Minimal expression of NSC markers in MSCs. Undifferentiated T-MSCs and undifferentiated BM-MSCs show low IF staining intensity for the neural stem cell markers MSI1, Nestin, and SOX1. DAPI serves as a nuclear counterstain.

Additional Fig. S2

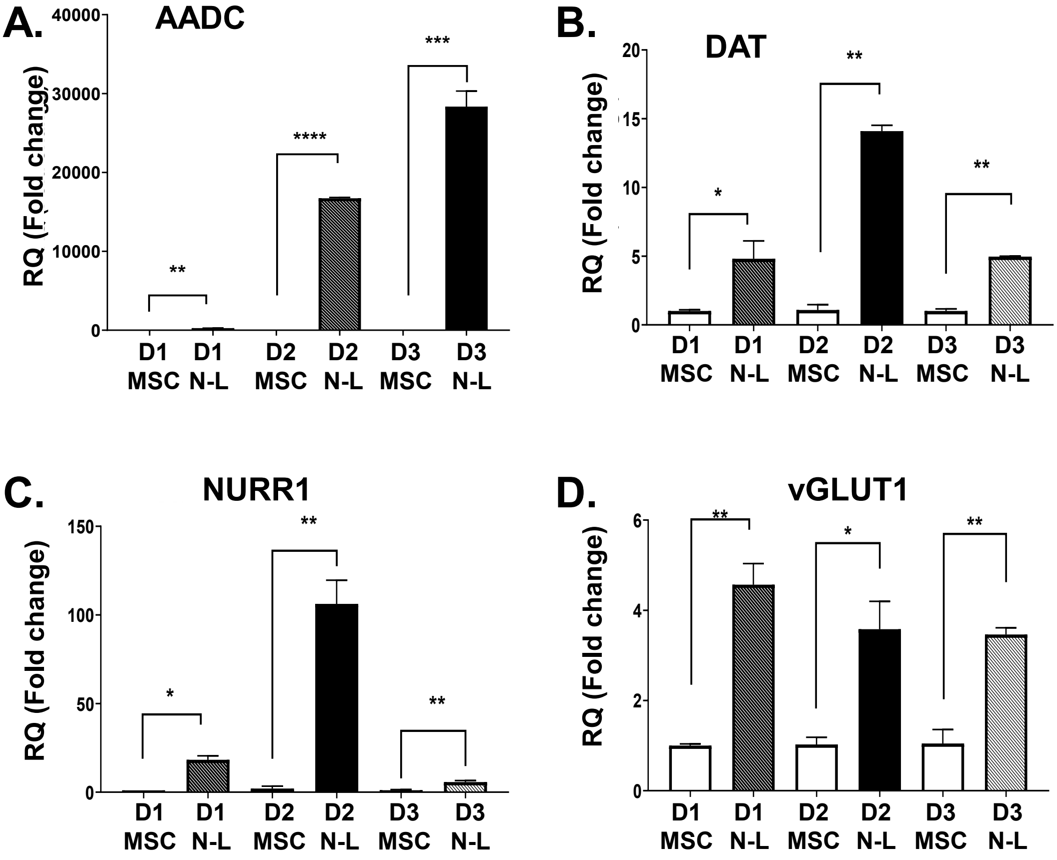

Supplementary Figure 2: Expression of dopaminergic and glutamatergic markers in our neuron-like cells. qRT-PCR data show a major increase of the dopaminergic neuron markers by day 28 Neuron-like cells: **A.** AADC, **B.** DAT, **C.** NURR1, and **D.** the glutamatergic marker vGLUT1. Data shown as mean ± SEM. Asterisks signify * $p<0.05$, ** $p<0.01$, *** $p<0.001$, **** $p<0.0001$. Data are shown from 3 representative donors (D1, D2, D3).

Additional Fig. S3

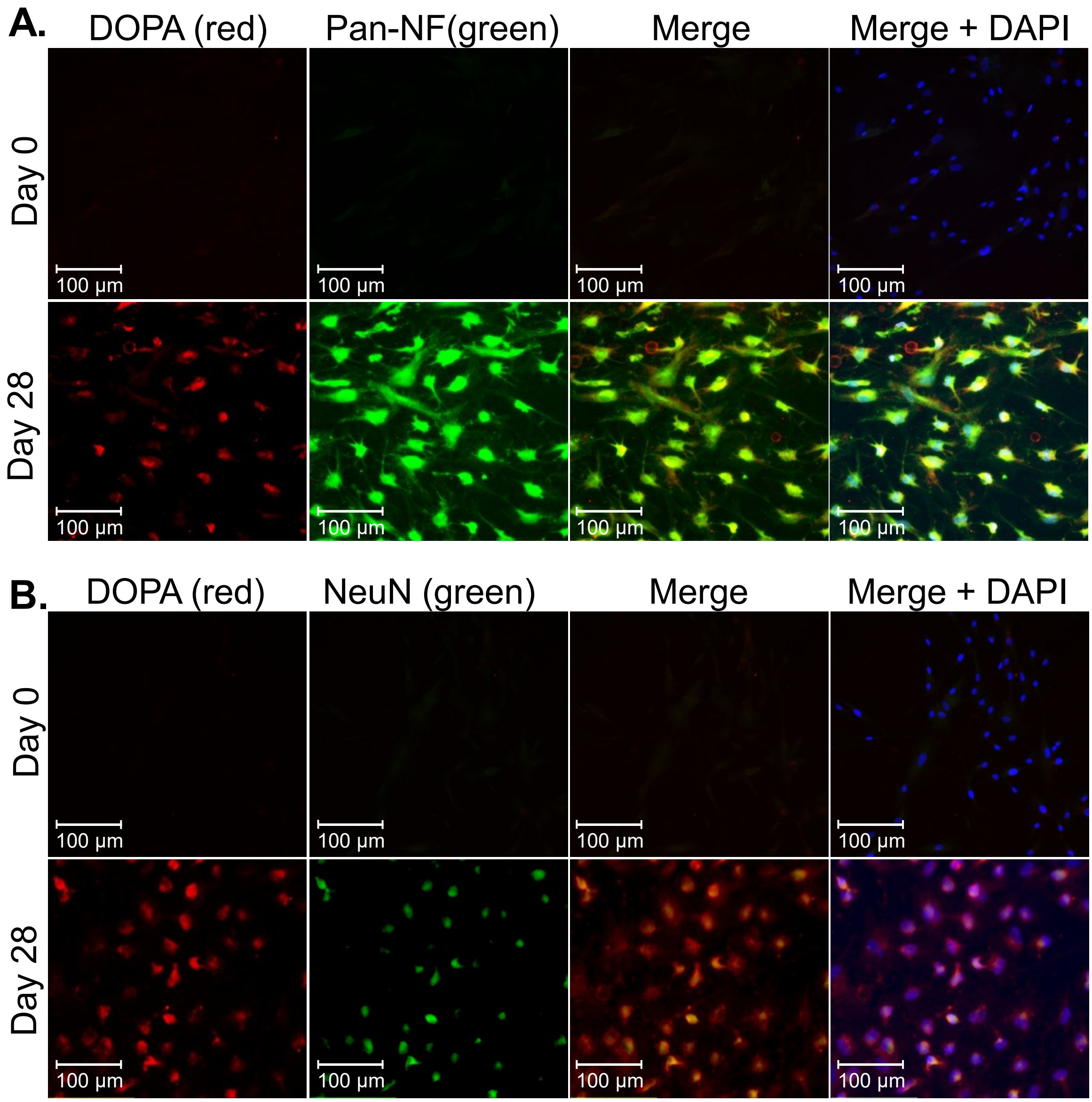

Supplementary Figure 3: Dopamine and neuronal markers in T-MSC derived neuron-like cells after 28 days of differentiation. **A.** Co-immunostaining show that dopamine (DOPA; red) is expressed in 15% of our cells and is co-expressed with the neuronal marker Pan-Neurofilament (Smi312; green), and **B.** the post-mitotic neuron marker NeuN (green). We found that the cells tend to grow in clusters in the culture well, therefore, figures show representative images of positive area. DAPI marks nuclei (blue). Undifferentiated MSCs were used as controls (Day 0).

Additional Fig. S4

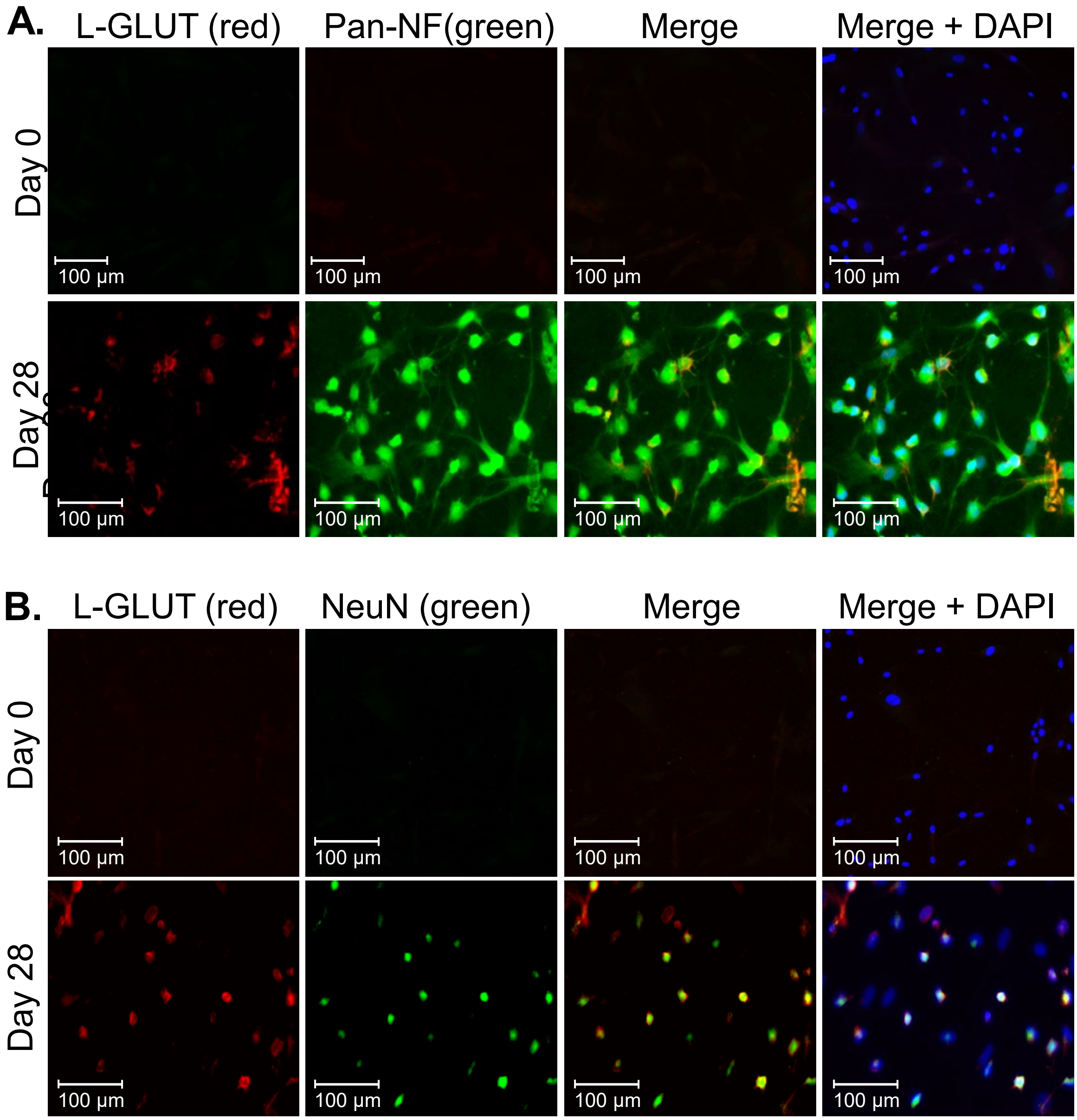

Supplementary figure 4: Glutamatergic neuronal markers in T-MSC-derived neuron-like cells. **A.** Co-immunostaining show that L-glutamate (L-GLUT; red) is expressed in 20% of our cells and is co-stained with the neuronal marker Pan-Neurofilament (Smi312), and **B.** with the post-mitotic neuron marker NeuN (green). Nuclei were counterstained by DAPI (blue). Undifferentiated MSCs were used as controls (Day 0). Images show positive clusters.
