# Supplementary material for: Direct differentiation of tonsillar biopsy-derived stem cells to the neuronal lineage
Source: Cell Mol Biol Lett. 2021 Aug 18;26:38. doi: 10.1186/s11658-021-00279-4 (PMC8371824; doi:10.1186/s11658-021-00279-4)
Supplement: Supplementary file 2 — Additional file 2: Table S1. Donor information. [file 11658_2021_279_MOESM2_ESM.pdf]

A.

**Donor number**	**Male/Female**	**Age (years)**
Donor 1	M	7
Donor 2	F	27
Donor 3	M	30
Donor 4	F	4
Donor 5	F	31

B.

Age (years)	Female	Male	total
3-7	1	1	2
20-35	2	1	3
Total	3	2	5

Additional Table 1: Donor information.
