# Supplementary material for: Direct differentiation of tonsillar biopsy-derived stem cells to the neuronal lineage
Source: Cell Mol Biol Lett. 2021 Aug 18;26:38. doi: 10.1186/s11658-021-00279-4 (PMC8371824; doi:10.1186/s11658-021-00279-4)
Supplement: Supplementary file 3 — Additional file 3: Table S2. List of Real-time RT-qPCR Primers. [file 11658_2021_279_MOESM3_ESM.pdf]

Primer	Forward 5'-3'	Reverse 5'-3'
Nestin	GCTACTGAAAAGTTCCAGCTGG	CTGAAAGCTGAGGGAAGTCTTG
DCX	GGAAGCACAAGGACCTGTAC	TAGGCTTGGATTTGTACTCTGG
ACTA2 /α SMA	GTTACTACTGCTGAGCGTGAGA	GATGCTGTTGTAGGTGGTTTCA
Beta3-tub	CATCTTTGGTCAGAGTGGGG	GTCGCAGTTTTCACACTCCT
NEFL	GAGATTGCAGCTTACAGGAAAC	GGTGGACATCAGATAGGAGCT
MAP2	TGGAGGTGGTAATGTCAAGATTG	GGGGACTGTGTAATGATCTCAG
PSD95 (DLG4)	CCTGGAGAATGTGCTAGAGATT	GGTAGATCTCCTCAAAGCTGTC
MSI 1	GCCATGCTGATGTTC GACA	CTACGATGTCCTCGCTCTCA
CD73	CAG TAC CAG GGC ACT ATC TGG	AGT GGC CCC TTT GCT TTA AT
CD90	ATGAACCTGGCCATCAGCA	GTGTGCTCAGGCACCCC
CD105	CCA CTA GCC AGG TCT CGA AG	GAT GCA GGA AGA CAC TGC TG
SOX2	CAAGGAGAGGCTTCTTGCTGA	CACAGAGATGGTTCGCCAGT
RPLP0	CAGCAAGTGGGAAGGTGTAATCC	CCCATTCTATCATCAACGGGT
GAPDH	CAAGATCATCACGAATGCCTC	GCATGGACTGTGGTCATGAGTC
AADC	GACTGTCTCAGTGCCGAGTG	TGTCAGAGGTGAAAAATGCTGG
DAT	GCCATGGGTGGTATGGAGTC	GTAGATGCCACCGTTGGTGA
NURR1	GGGACAAGCATGTTGATTCTAGG	AGCTGCTGCATGCAAGTTTT
vGLUT1	AGCTGGGATCCAGAGACTGT	CCGAAAACTCTGTTGGCTGC
GLS	GCTATGGACATGGAACAGCG	TATTCCACCTGTCCTTGGGGA
vAChT	GGCATAGCCCTAGTCGACAC	CTCATCAAGCAGCACATCGC
ChAT	AGCGCTCCGGGTAGATTCTG	ACCCGACTGGAGAAAGCAAGC
GAD65	GGGGACTACCGGGTTTGAAG	AATCACTGGAGCCACCTTCG
vGAT	CATTGCGACGACCTCGACTT	AACATGCCCTGGATGGCGTT
DBH	CACTGCCTATTTTGCGGACG	CTCCGGTTCGGGGATATTGG

Table 2: List of Real-time RT-qPCR Primers
