# Supplementary material for: Direct differentiation of tonsillar biopsy-derived stem cells to the neuronal lineage
Source: Cell Mol Biol Lett. 2021 Aug 18;26:38. doi: 10.1186/s11658-021-00279-4 (PMC8371824; doi:10.1186/s11658-021-00279-4)
Supplement: Supplementary file 4 — Additional file 4: Table S3. Antibodies list. [file 11658_2021_279_MOESM4_ESM.pdf]

Primary antibodies	Dilution Factor	Supplier
Smi-312 /Pan neurofilament	1:1000	Cell Signaling
Tuj1/β3-tubulin	1:200	Cell Signaling
MAP2	1:50	Cell Signaling
Synaptophysin	1:200	Cell Signaling
Neurofilament-L (NEFL)	1:100	Cell Signaling
PSD95	1:250	Cell Signaling
Musashi (MSI1)	1:500	Invitrogen
Nestin	1:2000	Cell Signaling
CD73-PE	1: 50	Biolegend
CD90-Alexa647	1:50	Biolegend
CD105-Alexa488	1:50	Biolegend
KI67	1:100	Invitrogen
Dopamine	1:200	Abcam
L-Glutamate	1:100	Sigma-Aldrich
Cyclin A2	1:100	Cell Signaling
NeuN/Rbfox3	1:500	Novus

Table 3: Antibodies list
